# Collective Influence Algorithm to find influencers via optimal percolation in massively large social media

**DOI:** 10.1038/srep30062

**Published:** 2016-07-26

**Authors:** Flaviano Morone, Byungjoon Min, Lin Bo, Romain Mari, Hernán A. Makse

**Affiliations:** 1 Levich Institute and Physics Department, City College of New York, New York 10031, NY, USA

## Abstract

We elaborate on a linear-time implementation of Collective-Influence (CI) algorithm introduced by Morone, Makse, Nature 524, 65 (2015) to find the minimal set of influencers in networks via optimal percolation. The computational complexity of CI is *O*(*N* log *N*) when removing nodes one-by-one, made possible through an appropriate data structure to process CI. We introduce two Belief-Propagation (BP) variants of CI that consider global optimization via message-passing: CI propagation (CI_P_) and Collective-Immunization-Belief-Propagation algorithm (CI_BP_) based on optimal immunization. Both identify a slightly smaller fraction of influencers than CI and, remarkably, reproduce the exact analytical optimal percolation threshold obtained in Random Struct. Alg. 21, 397 (2002) for cubic random regular graphs, leaving little room for improvement for random graphs. However, the small augmented performance comes at the expense of increasing running time to *O*(*N*^2^), rendering BP prohibitive for modern-day big-data. For instance, for big-data social networks of 200 million users (e.g., Twitter users sending 500 million tweets/day), CI finds influencers in 2.5 hours on a single CPU, while all BP algorithms (CI_P_, CI_BP_ and BDP) would take more than 3,000 years to accomplish the same task.

In ref. 1 we developed the theory of influence maximization in complex networks, and we introduced the Collective Influence (CI) algorithm for localizing the minimal number of influential nodes. The CI algorithm can be applied to a broad class of problems, including the optimal immunization of human contact networks and the optimal spreading of informations in social media, which are ubiquitous in network science^2^,^3^,^4^,^5^,^6^. In fact, these two problems can be treated in a unified framework. As we noticed in^1^, the concept of influence is tightly related to the concept of network integrity. More precisely, the most influential nodes in a complex network form the minimal set whose removal would dismantle the network in many disconnected and non-extensive components. The measure of this fragmentation is the size of the largest cluster of nodes, called the giant component *G* of the network, and the problem of finding the minimal set of influencers can be mapped to optimal percolation.

The influence maximization problem is NP-hard^7^, and it can be approximately solved by different methods. We showed in^1^ that the objective function of this optimization problem is the largest eigenvalue of the non-backtracking matrix (NB) of the network 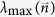, where 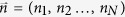 is the vector of occupation numbers encoding node’s vacancy (*n*_*i*_ = 0) or occupancy (*n*_*i*_ = 1). In^1^ we introduced the Collective Influence algorithm to minimize 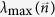. This algorithm is able to produce nearly optimal solutions in almost linear time, and performs better than any other algorithm with comparable, i.e. nearly linear, computational running time.

In this paper we describe an improved implementation of the original CI algorithm, which keeps the computational complexity bounded by *O*(*N* log *N*) even when nodes are removed one-by-one. This is made possible by the finite size of the Collective Influence sphere, which, in turn, allows one to use a max-heap data structure for processing very efficiently the CI values. The linear time implementation of CI is explained in Section *Implementing CI in linear time*.

In Section *CI propagation* we introduce a generalized version of the CI algorithm, which we name Collective Influence Propagation (CI_P_), that incorporates the information about nodes influence at the global level. Indeed, it can be seen as the limit version of CI when the radius ℓ of the ball is sent to infinity. The CI_P_ algorithm allows one to obtain a slightly better solution to the problem, i.e., a set of optimal influencers smaller than the one found by CI. Remarkably, it is able to reach the exact optimal percolation threshold in random cubic graphs, as found analytically by Bau *et al*.^10^. However, this augmented performance comes at the expense of increasing the computational complexity of the algorithm from *O*(*N* log *N*) to *O*(*N*^2^), when nodes are deleted one-by-one (the max-heap trick cannot be exploited in this case). The same quadratic running time pertains also to a Belief-Propagation-Decimation (BPD) algorithm recently suggested in ref. 12. Based on this observation, CI remains the viable option for a fast and nearly-optimal influencer search engine in massively large networks. Quantitatively, a network of 200 millions nodes can be fully processed by CI (using a radius ℓ = 2) in roughly 2.5 hours, while both CI_P_ and BPD would take a time of the order of 3,000 years to accomplish the task, as illustrated in Section *CI Propagation* and figures therein contained. In Section *Collective Immunization* we present yet another algorithm to solve the optimal influence problem, that we name CI_BP_ . The CI_BP_ algorithm is a belief-propagation-like algorithm inspired by the SIR disease spreading model, which provides as well nearly optimal solutions.

## Implementing CI in linear time

In this section we describe how to implement the CI algorithm to keep the running time *O*(*N* log *N*) even when the nodes are removed one-by-one. CI is an adaptive algorithm which removes nodes progressively according to their current CI value, given by the following formula:


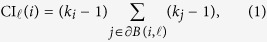


where *k*_*i*_ is the degree of node *i*, *B*(*i*, ℓ) is the ball of radius ℓ centered on node *i*, and ∂*B*(*i*, ℓ) is the frontier of the ball, that is, the set of nodes at distance ℓ from *i* (the distance between two nodes is defined as the number of edges of the shortest path connecting them).

At each step, the algorithm removes the node with the highest CI_ℓ_(*i*) value, and keeps doing so until the giant component is destroyed. A straightforward implementation of the algorithm consists in computing at each step CI_ℓ_(*i*) for each node *i*, and then removing the node with the largest CI_ℓ_ value. Despite its simplicity, this implementation is not optimal, as it takes a number of operations of order *O*(*N*^2^). However, the time complexity of the CI-algorithm can be kept at *O*(*N* log *N*) by using an appropriate data structure for storing and processing the CI values. The basic idea is that, after each node removal, we actually need to recompute CI just for a *O*(1) number of nodes, and find the new largest value O(log N) operations. This idea can be concretized through the use of a max-heap data structure.

**Figure 1:**
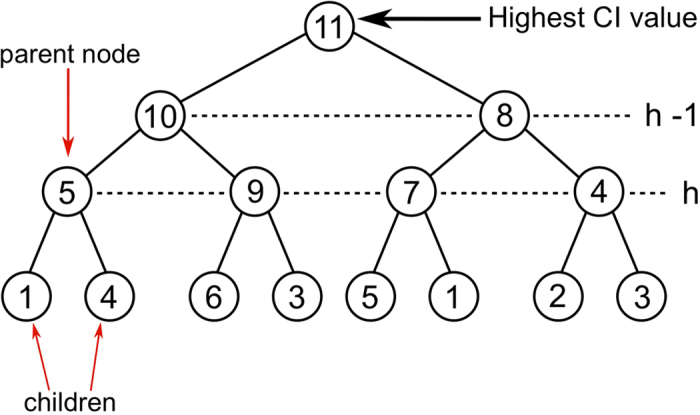
Max-heap data structure used to implement the CI algorithm. In the max-heap each parent node stores a CI value larger than the ones stored by its children. No ordering prescription is imposed to the nodes belonging to the same level *h* of the heap.

Before to delve into the details, let us recall the definition of a “heap”. A heap is a binary tree encoding a prescribed hierarchical rule between the parent node at level *h* and its children nodes at level *h* + 1, with no hierarchy among the children. In our specific case we use a heap with a max-heap rule, i.e., each parent node of the heap stores a CI value greater or equal to those of the children, but there is no order between the left child and the right one (see Fig. 1). The root node of the max-heap stores automatically the largest CI value.

One more concept is needed, i.e., the concept of “heapification”, which we shall be using often later on. Generally speaking, given a set of numbers *S* = {*x*_1_, …, *x*_*N*_}, the heapification of the set *S* is a permutation ∏ of the elements {*x*_∏(1)_, …, *x*_∏(*N* )_} satisfying the following max-heap property:


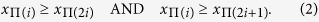


We call heapify(*i*) the function which heapifies the CI values in the sub-tree rooted on node *i*. The aim of this function is to down-move node *i* in the heap by swapping it with the largest of its children until it satisfies the max-heap property in the final location.

**Figure 2:**
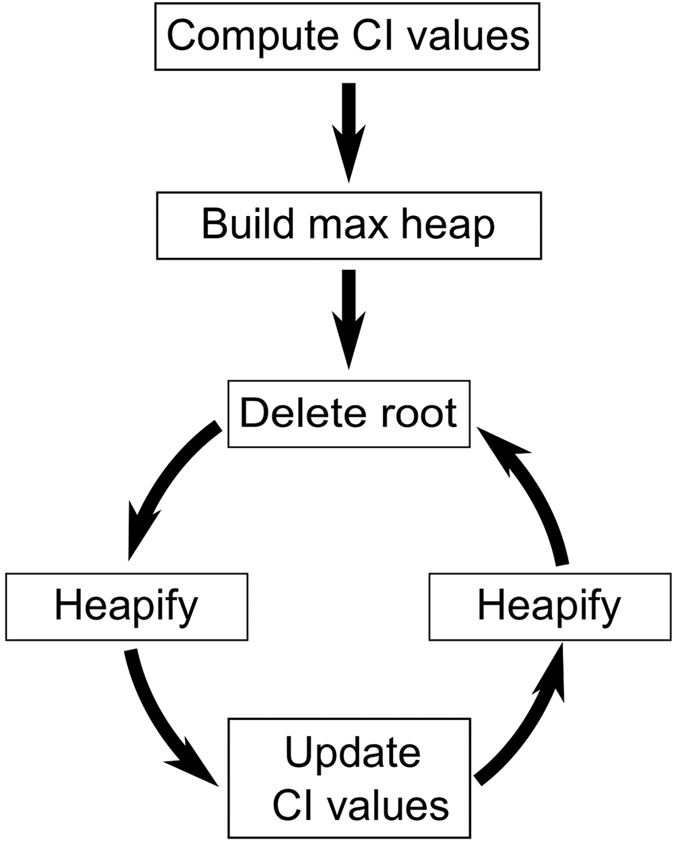
Flow of the CI algorithm. The first part of the algorithm, executed only once, consists of two steps: i) computing CI for each node, and ii) allocating the CI values in the max-heap. After that, the main loop of the algorithm follows, which consists of three steps: iii) removing the node with highest CI value along with the root of the heap; iv) heapifying the heap starting from the new root (see Step3); v) updating the CI values of the perturbed nodes, and heapifying the sub-trees rooted on each updated node. The loop ends when the giant component is destroyed.

Having defined the main tools we are going to use in the implementation, we can now discuss the flow of the algorithm schematized in Fig. 2 step by step.

### Step 1 - Computing CI

To compute the CI_ℓ_(*i*) value of node *i* according to Eq. (1) we have to find the nodes belonging to the frontier ∂*B*(*i*, ℓ) of the ball of radius ℓ centered on *i*. In an undirected network, nodes *j* ∈ ∂*B*(*i*, ℓ) can be found by using a simple breadth-first-search up to a distance ℓ from the central node *i*. In practice, first we visit the nearest neighbours of node *i*, which, of course, belong to ∂*B*(*i*, 1). Then we visit all the neighbours of those nodes not yet visited, thus arriving to ∂*B*(*i*, 2). We keep going until we visit all the nodes in ∂*B*(*i*, ℓ). At this point we use the nodes *j* ∈ ∂*B*(*i*, ℓ) to evaluate CI_ℓ_(*i*) using Eq. (1). When all the CI values {CI_ℓ_(1), …, CI_*ℓ*_(*N*)} have been calculated, we arrange them in a max-heap, as explained next.

### Step2 - Building the max-heap

We build the heap in a bottom-up fashion, from the leaves to the root. Practically, we first fill the heap with arbitrary values and then we heapify all the levels starting from the lowest one. In this way the root stores automatically the largest CI value.

### Step3 - Removal

**Figure 3:**
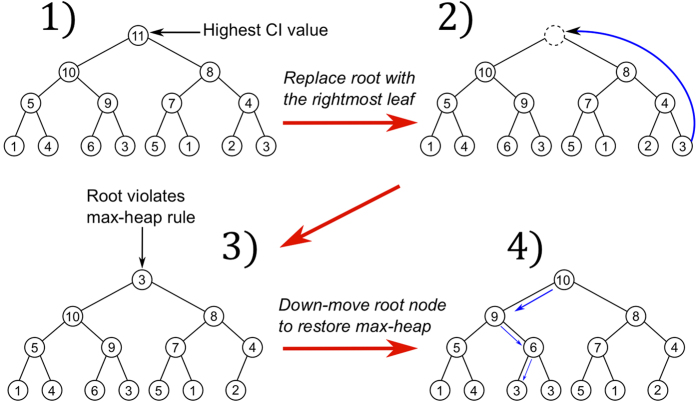
Schematic representation of how to update the heap. In step 1) the root node storing the highest CI value, node 11 in this case, is removed. In step 2) the root is replaced by the rightmost leaf of the heap, that is node 3 in this example. In step 3) the new heap does not satisfy the max-heap rule. In step 4) the heapification starting from the root restores the max-heap structure. The heapification down-moves progressively node 3 in the heap by swapping it with the largest of its children nodes, until it does satisfy the max-heap property in the final location.

We remove from the network the node having the largest CI value, and we decrement by one the degrees of its neighbors. Since the largest CI value is stored in the root of the max-heap, then, after its removal, the root has to be replaced by a new one storing the new largest CI value. The easiest way to do this consists in replacing the old root with the rightmost leaf in the last level of the heap, decreasing the size of the heap by one, and heapifying the heap starting from the new root, as shown schematically in Fig. 3.

### Step4 - Updating CI values

**Figure 4:**
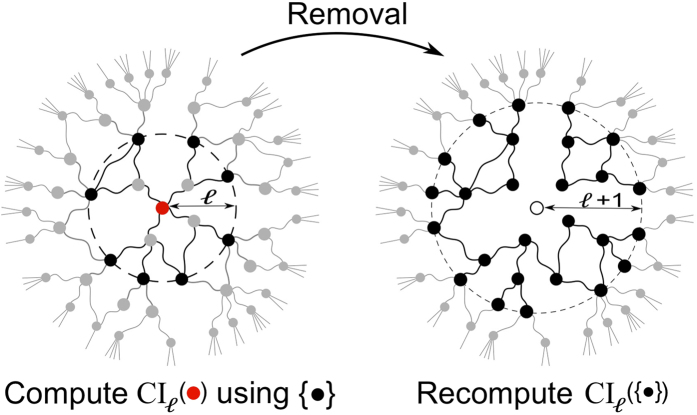
**Left panel:** the CI of the red node at the level ℓ is computed using the nodes on the boundary of the ball of radius ℓ centered on the red node. **Right panel**: the removal of the red node perturbs the CI values of nodes located up to a distance ℓ + 1 from it. Accordingly, only the CI values of these nodes, i.e. the black ones, must be updated before the next removal.

Removal of a node perturbs the CI values of other nodes, which must be recomputed before the next removal. The nodes perturbed by the removal are only the ones placed at distances 1, 2, …, ℓ, ℓ + 1 from the removed one. In other words, only the nodes inside the ball B(*i*, ℓ + 1) change their CI values when *i* is removed, while the others remain the same (see Fig. 4).

The CI values of nodes on the farthest layer at ℓ + 1 are easy to recompute. Indeed, let us consider one of this node and let us call *k* its degree. After the removal of the central node its CI value decreases simply by the amount *k* − 1. For nodes in the other layers at distance 1, 2, …, ℓ, the shift of their CI values is, in general, not equally simple to assess, and we need to use the procedure explained in Step1.

When we modify the CI value stored in a node of the heap, it may happen that the new heap does not satisfy the max-heap rule, and hence we have to restore the max heap-structure after each change of the CI values. First of all we note that, under the hypothesis that the structure around the removed node is locally tree-like, the new CI values of the surrounding nodes can only be smaller than their old values (for ℓ ≤ 2 this is always true even without the tree-like hypothesis). Consequently, we need to heapify only the sub-trees rooted on those nodes. We stress that the order of the update and heapification operations is important: each node update must be followed by the corresponding heapification, before updating the next node.

### Step5 - Stopping the algorithm

**Figure 5:**
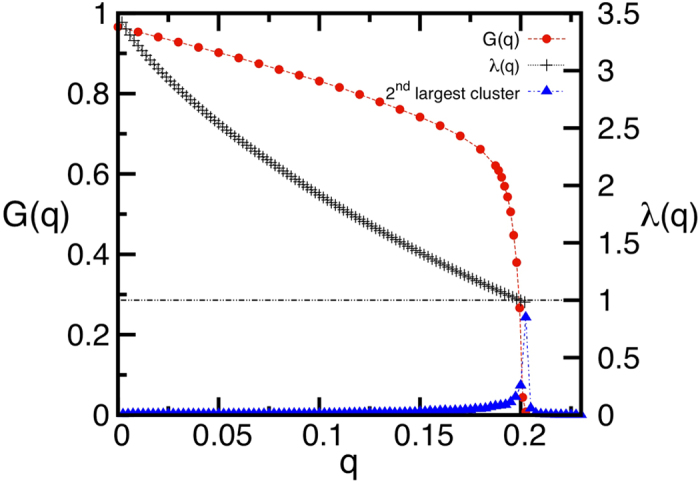
Giant component *G*(*q*) (red dots) computed with CI, second largest cluster (blue triangles), and the eigenvalue *λ*(ℓ; *q*) (black crosses) as given by Eq. (3), as a function of the removed nodes *q*. Here we used an ER network of 10^6^ nodes, average degree 〈*k*〉 = 3.5, and radius of the CI sphere equal to ℓ = 5. The eigenvalue *λ*(*q*) reaches one when the giant component is zero, as marked also by the peak in the size of the second largest cluster. In this plot the size of the second largest cluster is magnified to make it visible at the scale of the giant component.

**Figure 6:**
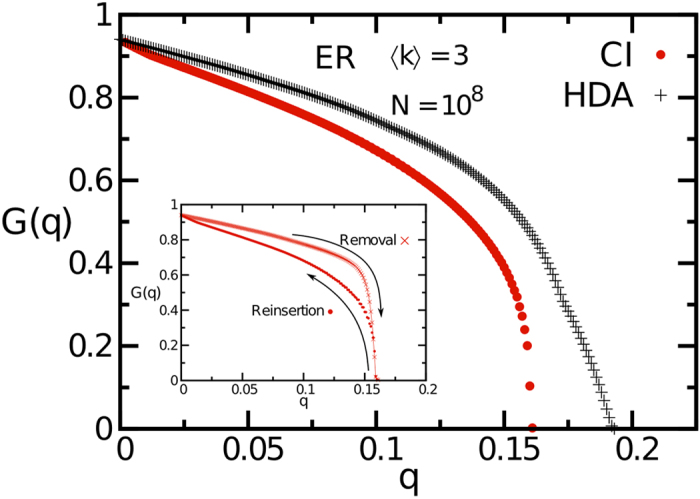
Giant component *G*(*q*) as a function of the fraction of nodes *q* removed using CI algorithm (red dots) on a ER network of 10^8^ nodes. The result obtained by using CI is compared with the one obtained by using the HDA (high degree adaptive) strategy (black crosses), one of the few strategies which is adaptive and linear in algorithmic time. Indeed, the max-heap trick can be used also for other adaptive algorithms having the same properties of CI, such as HDA (which corresponds basically to the ℓ = 0 limit of the CI algorithm). Consequently, HDA has the same running time of its non-adaptive version, i.e., the simple high-degree centrality. However, exploiting a max-heap is not feasible for general adaptive algorithms, like CI_P_ or BPD. Therefore, since we are unable to keep their running time linear in the system size when nodes are removed one-by-one, we cannot apply them on the large network instance used in this example, as their running time for this network is about 10^3^ years. In the inset we show the giant component *G*(*q*) before and after the application of the reinsertion method discussed in Sec. *Implementing CI in linear time* at Step6.

To decide when the algorithm has to be terminated we use a very simple method, which allows one to avoid checking when the giant component *G* vanishes. The idea is to monitor the following quantity after each node removal:


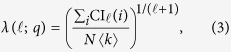


where 〈*k*〉 is the average degree of the network for *q* = 0. Equation (3) gives an approximation of the minimum of the largest eigenvalue of the non-backtracking matrix when *Nq* nodes are removed from the network^1^. For *q* = 0, it is easy to show that, for tree-like random graphs, *λ*(ℓ; 0) = *κ* − 1, where *κ* = 〈*k*^2^〉/〈*k*〉. Removing nodes decreases the eigenvalue *λ*(ℓ; *q*), and the network is destroyed when lim_ℓ→∞_*λ*(ℓ; *q* = *q*_*c*_) = 1. Practically we cannot take the limit ℓ → ∞, but for ℓ reasonably large, the relaxed condition *λ*(ℓ; *q* = *q*_*c*_) = 1 works pretty well, as we show in Fig. 5. Therefore, we can stop the algorithm when *λ*(ℓ; *q*) = 1. The advantage of using Eq. (3) is that it can be updated on runtime at nearly no additional computational cost, and therefore does not require additional *O*(*N*) calculations (per node removal) needed to compute the giant component. Figure 6 shows the giant component attacked by CI and high-degree adaptive in a ER network of 100 million nodes.

**Figure 7:**
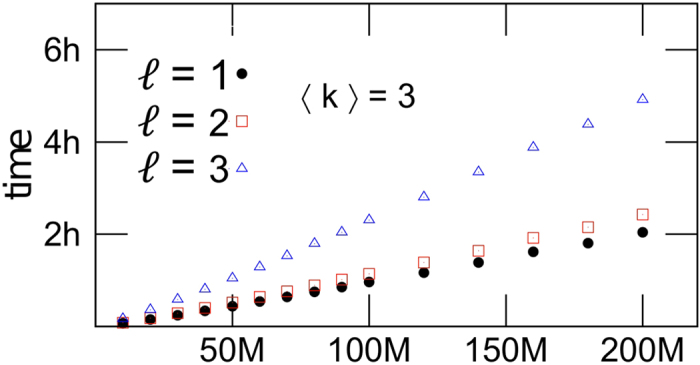
Running time of the CI algorithm (including the reinsertion step) for ER random graphs of average degree 〈*k*〉 = 3, as a function of the network size, and for different values of the radius ℓ of the ball. (To generate very large ER random graphs we used the algorithm of ref. 8). For a graph with 0.2 billion nodes the running time is less than 2.5 hours with ℓ = 2 and ∼5 hours with ℓ = 3.

The running time of CI algorithm is *O*(*N* log *N*). In fact, Step1 and Step2 take both *O*(*N*) operations and they are performed only once. Step3 and Step4 take each at most *O*(log *N*) operations and they are repeated *O*(*N*) times. Therefore the algorithm takes *O*(*N* log *N* + *N*) ∼ *O*(*N* log *N*) operations. To check the (*N* log *N*) scaling of the CI algorithm we performed extensive numerical simulations on very large networks up to *N* = 2 × 10^8^ nodes. The results shown in Fig. 7 clearly confirm that CI runs in nearly linear time.

### Step6 - Reinsertion

We conclude this section by discussing a refinement of CI algorithm, which we use to minimize the giant component in the phase *G* > 0. This is useful when it is impossible to reach the optimal percolation threshold (where *G* = 0), but one still wants to minimize *G* using the available resources, i.e., the maximum number of node removals at one’s disposal. The main idea is based on a reinsertion method, according to which nodes are reinserted in the network using the following criterion. We start from the percolation point, where the network is fragmented in many clusters. We add back in the network one of the removed node, chosen such that, once reinserted, it joins the smallest number of clusters. Note that we do not require that the reinserted node joins the clusters of smallest sizes, but only the minimum number of clusters, independently from their sizes. When the node is reinserted we restore also the edges with its neighbors which are in the network (but not the ones with neighbors not yet reinserted, if any). The procedure is repeated until all the nodes are back in the network. When implementing the reinsertion, we add back a finite fraction of nodes at each step. In our simulations we reinserted 0.2% of nodes at each step. Moreover, we observed that using a fraction smaller than 0.2% does not change the results. In the inset of Fig. 6 we show the giant component *G*(*q*) as a function of the fraction of removed nodes *q* before and after the reinsertion step.

## CI propagation

In this section we present the CI-propagation algorithm (CI_P_), which extends the CI algorithm to take into account the global information beyond the local CI sphere. However, the main idea of CI_P_ remains the same, i.e., minimizing the largest eigenvalue of the non-backtracking (NB) matrix^1^. Indeed, CI_P_ is obtained asymptotically from CI_ℓ_ as ℓ → ∞.

The NB is a non-symmetric matrix and it has different right and left eigenvectors. As we will see the right and left eigenvectors corresponding to the largest eigenvalue provides two different, yet intuitive, notions of node’s influence. The left eigenvector 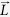 is a vector with 2*M* entries *L*_*i*→*j*_, where *M* is the total number of links, that satisfies the following equation:


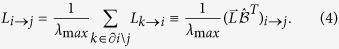


A similar equation holds for the right eigenvector 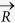:


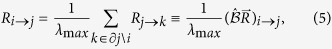


where 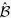 is the NB matrix. Both left and right eigenvectors can be thought of as two sets of messages traveling along the directed edges of the network. This becomes more apparent if we transform Eqs (4, 5) in dynamical updating rules for the messages *L*_*i*→*j*_ and *R*_*i*→*j*_ as:


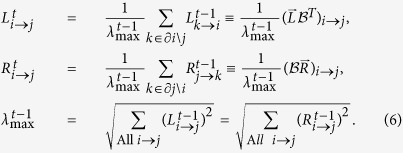


The interpretation of Eq. (6) is the following. For each directed edge *i* → *j*, the message 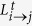 at time *t* from *i* to *j* is updated using the messages 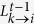 incoming into node *i* at time *t* − 1, except the message 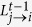. Therefore, the left message 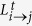 represents the amount of information received by node *i* from its neighbours, other than *j*. On the contrary, the right message *R*_*i*→*j*_ is updated using the sum of the outgoing messages from node *j* to nodes *k* other than *i*, and thus it measures the amount of information sent by node *j* to its neighbours, other than *i*.

Now we come to the problem of minimizing the largest eigenvalue *λ*_max_ of the NB matrix 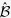 by removing nodes one-by-one. Let us consider the non-backtracking matrix 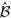 of the network. When we remove a node from the network, the NB matrix 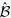 changes as a consequence of the node deletion. Let us call 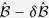 the NB matrix of the network with one node less. Similarly, also the right and left eigenvectors of 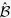 change after the node removal. We call 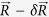 and 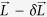 the right and left eigenvectors of the perturbed NB matrix 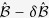. Then, the eigenvalue equation for the matrix 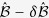 reads:


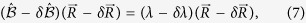


where *λ* − *δλ* is the new eigenvalue after the node removal. We also note that *δλ* ≥ 0, and that the entries of the matrix 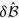 are non-negative. Assuming that all the terms 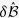, 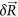 and *δλ* can be treated as small, so that we can neglect contributions of order 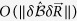 and 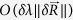, we obtain the following simpler eigenvalue equation:


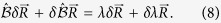


Now, by scalar multiplying on the left both sides of Eq. (8) by the left eigenvector 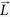 of the NB matrix 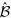, we obtain the following equation for the eigenvalue shift *δλ* due to removal of a node:


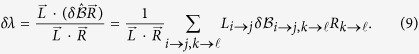


Next, we notice that the matrix 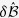 has non-zero components only on the pairs of non-backtracking edges (*i* → *j*, *k* → ℓ) containing the removed node in any position. If we call *i* the removed node, then 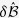 has non-zero components, equal 1, only for the following pairs of non-backtracking edges:


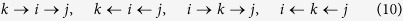


for all *j*, *k* ∈ ∂*i*. Taking into account only the contributions in Eq. (10) to evaluate the sum on the r.h.s of Eq. (9), we find:


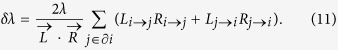


From Eq. (11) is clear that the node *i* which decreases the most the largest eigenvalue *λ* of the NB matrix 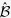 after its removal, is the one that maximizes the sum on the r.h.s. of Eq. (11). We call this sum the Collective Influence Propagation of node *i*, which we define as:


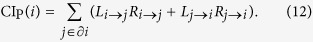


The quantity CI_P_(*i*) combines both the information received and the information broadcasted by node *i*. The interpretation of this quantity comes directly from the recursive Eqs (4) and (5). Indeed, if we plug the recursive Eq. (4) for *L*_*i*→*j*_ into (12), and we keep iterating ℓ times, we obtain the sum of all the messages *L*_→Ball(*i*, ℓ)_ incoming into the ball of radius ℓ centered on *i*. Similarly, by plugging Eq. (5) for *R*_*i*→*j*_ into (12) and iterating ℓ times, we obtain the sum of all the messages *R*_→Ball(*i*, ℓ)_ outgoing from the ball of radius ℓ centered on *i* (analogous interpretations hold for *L*_*i*→*j*_ and *R*_*i*→*j*_). With a bit of verbal playfulness, we could say that Eq. (12) quantifies both the “IN-fluence” and the “OUT-fluence” of node *i*.

Having defined the main quantity of the CI_P_ algorithm, we move to explain the few simple steps to implement it.

1. Start with all nodes present and iterate Eq. (6) until convergence.
2. Use the converged messages *L*_*i*→*j*_ and *R*_*i*→*j*_ to compute the CI_P_(*i*) values for each node *i*.
3. Remove node i^*^ with the highest value of CI_P_(i^*^) and set to zero all its ingoing and outgoing messages.
4. Repeat from 2) until *λ*_max_ = 1.

**Figure 8:**
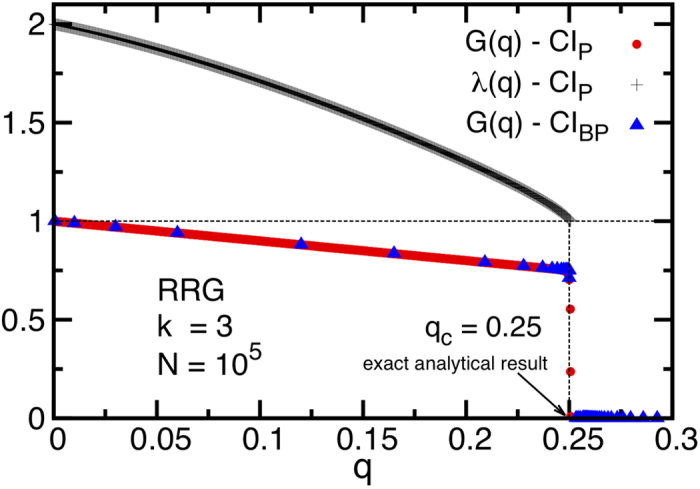
Giant components *G*(*q*) computed with *σi* = 1 (red dots) and CI_BP_ (blue triangles) algorithms, and the eigenvalue *λ*(*q*) (black crosses) computed with CI_P_, as a function of the removed nodes *q*, in a Random Regular Graph of 10^5^ nodes, and degree *k* = 3. The vertical line at *q* = 0.25 = *q*_*c*_ marks the position of the analytical exact optimal value of the percolation threshold^10^.

**Figure 9:**
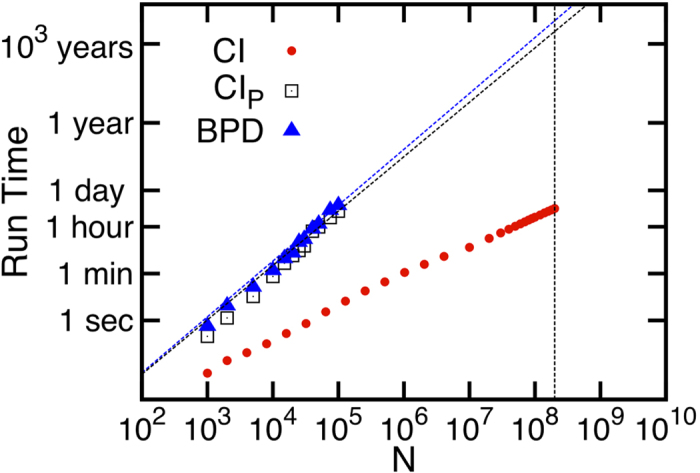
Running time of CI (red dots) at level ℓ = 3, CI_P_ (black squares), and the BPD algorithm of ref. 12 (blue triangles), as a function of the network size *N*, for ER networks of average degree 〈*k*〉 = 3. The CI algorithm is the only one that scales almost linearly with the system size, while both BP algorithms, CI_P_ and BPD, scale quadratically with the network size *N*. The vertical dashed line is at *N* = 2 × 10^8^: for this network size, the running time of CI at level ℓ = 3 is roughly 5 hours (and ∼2.5 hours for ℓ = 2), while both CI_P_ and BPD would take a time of ∼3,000 years to accomplish the same task. (To measure the running time of CI_P_ and BPD we used the same number of iterations of the messages. In all three algorithms nodes were removed one-by-one. Data are in log−log scale.) To draw the curve corresponding to the BPD algorithm we used the original source-code provided by the authors of ref. 12 (power.itp.ac.cn/zhouhj/codes.html).

The CI_P_ algorithm produces better results than CI. As we show in Fig. 8 for the case of a random cubic graph, CI_P_ is able to identify the optimal fraction of influencers, which is known analytically to be *q*_*c*_ = 1/4^10^. Unfortunately the CI_P_ algorithm has running time *O*(*N*^2^) and thus cannot be scaled to very large networks, as we show in Fig. 9, where we also compare with the time complexity of the BPD algorithm of ref. 12 and with the original CI algorithm. We also note that in the implementation of CI_P_ there is no need to compute explicitly the giant component, and the algorithm is terminated when the largest eigenvalue of the NB matrix equals *λ*_max_ = 1.

We close this section by noticing that CI_P_ is a parameter-free algorithm, i.e., it does not require any fine tuning and can be applied straight away thanks to its low programming complexity. The introduction of tunable parameters in the algorithm may improve its performance, but would not reduce its running time. Furthermore, the quasi-optimal performance of CI_P_ for finding minimal percolation sets in small systems in Fig. 8 leaves little room for improvement, and so we do not develop the algorithm further.

## Collective Immunization

In this section we formulate the optimal percolation problem as the limit of the optimal immunization problem in the SIR –Susceptible-Infected-Recovered– disease spreading model^11^, and we present the Collective Immunization algorithm, or CI_BP_, based on Belief Propagation.

According to the SIR model, a variable 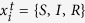 encodes the state of each node *i* at time step *t*. A node in a state *x*_*i*_ = *I* stays infected for a finite time, and in this state, it may infect a neighboring node *j* if *x*_*j*_ = *S*. After the infectious period, the infected node *i* recovers. Nodes in state *R* stay in *R* forever, being immune to further infection. Thus in the long time limit, the disease state 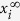 of any node *i* is either *R* or *S*. In this limit one can compute the marginals of *x*^∞^ on any node, knowing the initial state *x*^0^, in a ‘message passing’ manner. The message that node *i* passes to node *j* is the probability 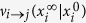 that node *i* ends in state 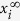 knowing it starts in state 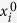, assuming that node *j* is absent.

According to the dynamic rule of SIR model, we have the following set of relations:


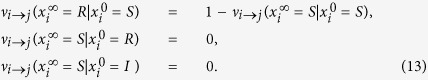


Therefore, it is clear that the knowledge of the sole 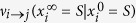 is enough to reconstruct the long time limit of the marginal of 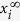. Next, we assume that each node is initially infected with probability *γ*, i.e., at time 0 a randomly chosen set of *γN* sites are infected. We also introduce a binary variable *n*_*i*_ for each node *i*, taking values *n*_*i*_ = 0 if node *i* is immunized (i.e. removed in the language of optimal percolation), and *n*_*i*_ = 1 if it is not (i.e. present). For a locally tree-like network where the interactions satisfy the cluster decomposition property (i.e. nodes far apart in the tree do not interfere), the probabilities (messages) received by node *i* from its neighbors *j* can be considered as uncorrelated. This allows one to calculate self-consistently the messages through the following equations:


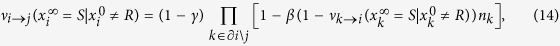


where *β* is the transmission probability of the disease (or the spreading rate). The optimal percolation problem is found in the limits *γ* = 1/*N* → 0 and *β* → 1.

The marginal probability that node *i* is eventually susceptible given that node *i* is not one of the immunizators is obtained through:


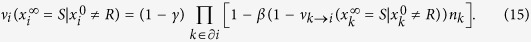


From now on we drop the argument in the probabilities ν_*i*→*j*_ and ν_*i*_, and we simply write 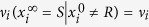.

The best immunization problem amounts to find the minimal set of initially immunized nodes that minimizes the outbreak size 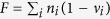. This problem can be equivalently solved by minimizing the following energy (or cost) function:


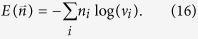


The energy function in Eq. (16) has the virtue of describing a pairwise model, and therefore is easier to treat. Indeed, substituting (15) into (16) one can rewrite the energy function as:


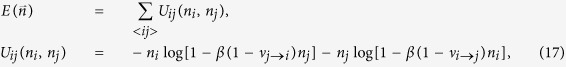


where we drop an useless constant term. We found useful to make the following change of variables:


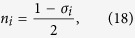


so that *σ*_*i*_ = 1 when node *i* is removed or immunized, and *σi* = −1 when it is present or not immunized. The minimum of the energy function (17) can be found by solving the following equations:


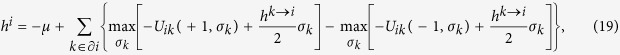



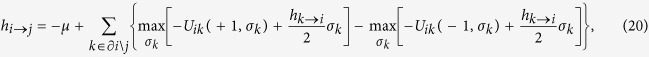


where the variable *h*_*i*_ is the log-likelihood ratio:


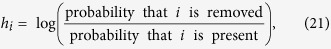


and *μ* is a parameter (chemical potential) that can be varied to fix the desired fraction of removed nodes *q*. The value of *σ*_*i*_ is related to *h*_*i*_ via the equation:


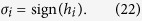


Equations (14), (19), (20) and (22) constitute the full set of equations of the immunization optimization problem, which, for *γ* = 0 and *β* = 1, is analogous to optimal percolation since, in this case, the best immunizators are those that optimally destroy the giant connected component. These equations can be solved iteratively as follows:

- Choose a value for *μ*, *γ*, *β*, and initialize all the state variables *σ*_*i*_ and *h*^*i*→*j*^ to random values.
- Then iterate Eqs (14) until convergence to find the values of ν_*i*→*j*_.
- Then iterate Eqs (20) until convergence to find the values of *h*_*i*→*j*_.
- Compute the new *h*^*i*^ using (19), and the the new state *σ*^*i*^ of node *i* via Eq. (22).
- Repeat until all the fields {*h*_*i*_} have converged.

In cases where the equations (20) do not converge, we use the reinforcement technique^9^. Once a solution to the equations has been found, the configuration 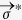 is the output of the algorithm: if 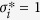 the node is removed, and if 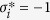 it is present. The CI_BP_ algorithm has the same performance as the CI_P_ algorithm, as we show for the case of random cubic graphs in Fig. 8, reproducing the exact result of ref. 10 for small system size and leaving virtually no room for improvement for these systems. However, while it improves over CI, it suffers the same deficiency for large systems as CI_P_ and BDP since it is a quadratic algorithm which can be applied only to small networks.

We conclude this section by emphasizing that all the algorithms discussed in this work leverage on the assumption about the locally tree-like structure of the network, while real networks may violate this hypothesis. Nonetheless, the tree-like approximation, also known as the Bethe ansatz, is a very good mean-field theory for complex networks, and corrections due to the presence of loops of finite length may be, in principle, accommodated systematically. Indeed, it would be very interesting and useful to develop further, for example, the theory of optimal percolation by including loop corrections, following the theoretical lines of, e.g., refs 13, 14, 15, 16.

## Conclusions

We have shown how to implement the CI algorithm introduced in ref. 1 in nearly linear time when nodes are removed one by one. This is possible thanks to the finite radius ℓ of the CI sphere, which in turn allows one to process the CI values in a max-heap data structure.

Moreover, we have introduced CI_P_ , a modified CI algorithm taking into account the global rearrangement of the CI values after each node removal, and, in this respect, it corresponds to the ℓ → ∞ limit of CI. We have also presented CI_BP_ , a new algorithm to solve the optimal percolation problem, which blends the dynamics of the SIR disease spreading model with message passing updating rules. The analysis of these algorithms (including BDP as well) reveals that the improvements over CI are small and, more importantly, they are made at the expense of increasing the computational complexity from linear (CI) to quadratic (BP) in the system size *N*, rendering BP unfit for large datasets.

Therefore, CI remains the viable option of a nearly-optimal-low-complexity influencer search engine, which is applicable to massively large networks of several hundred million of nodes, while the global CI_P_ algorithm can still be used to find small corrections in small networks when time performance is not an issue. Furthermore, from a theoretical point of view, the simplicity of the CI analysis based on the NB eigenvalue remains as a good option for theoretical generalization of optimal percolation to more complicated topologies, as shown in^17^,^18^ for brain network of networks with interdependencies and other more complex applications that are being presently developed.

## Additional Information

**How to cite this article**: Morone, F. *et al*. Collective Influence Algorithm to find influencers via optimal percolation in massively large social media. *Sci. Rep.* **6**, 30062; doi: 10.1038/srep30062 (2016).
